# Neurotoxic Soluble Amyloid Oligomers Drive Alzheimer’s Pathogenesis and Represent a Clinically Validated Target for Slowing Disease Progression

**DOI:** 10.3390/ijms22126355

**Published:** 2021-06-14

**Authors:** Martin Tolar, John Hey, Aidan Power, Susan Abushakra

**Affiliations:** Alzheon, Inc., Framingham, MA 01701, USA; john.hey@alzheon.com (J.H.); aidan.power@alzheon.com (A.P.); susan.abushakra@alzheon.com (S.A.)

**Keywords:** Alzheimer’s disease, beta amyloid oligomers, ALZ-801, tramiprosate, aducanumab, lecanemab, gantenerumab, donanemab, ε4 allele of apolipoprotein E (APOE4)

## Abstract

A large body of clinical and nonclinical evidence supports the role of neurotoxic soluble beta amyloid (amyloid, Aβ) oligomers as upstream pathogenic drivers of Alzheimer’s disease (AD). Recent late-stage trials in AD that have evaluated agents targeting distinct species of Aβ provide compelling evidence that inhibition of Aβ oligomer toxicity represents an effective approach to slow or stop disease progression: (1) only agents that target soluble Aβ oligomers show clinical efficacy in AD patients; (2) clearance of amyloid plaque does not correlate with clinical improvements; (3) agents that predominantly target amyloid monomers or plaque failed to show clinical effects; and (4) in positive trials, efficacy is greater in carriers of the ε4 allele of apolipoprotein E (APOE4), who are known to have higher brain concentrations of Aβ oligomers. These trials also show that inhibiting Aβ neurotoxicity leads to a reduction in tau pathology, suggesting a pathogenic sequence of events where amyloid toxicity drives an increase in tau formation and deposition. The late-stage agents with positive clinical or biomarker data include four antibodies that engage Aβ oligomers (aducanumab, lecanemab, gantenerumab, and donanemab) and ALZ-801, an oral agent that fully blocks the formation of Aβ oligomers at the clinical dose.

## 1. Introduction

The neuropathological diagnosis of Alzheimer’s disease (AD) requires the presence of two core pathologies: extracellular beta amyloid (amyloid, Aβ) plaques and aggregated tau protein forming intra-neuronal neurofibrillary tangles (tau, NFT) [1,2]. While older clinical studies enrolled patients based only on a clinical diagnosis of AD, most current trials require a biomarker-based diagnosis of AD by demonstrating either a cerebrospinal fluid (CSF) signature consistent with AD that shows soluble amyloid and tau, or by amyloid positron emission tomography (PET) imaging that detects insoluble amyloid [3]. This approach introduces some heterogeneity since the level of tau pathology can be variable among study subjects. Recently, a more precise approach utilized PET imaging of both amyloid and tau pathologies and selection of a specific range and location of tau PET signals. Despite being a defining pathology of AD, the role of beta amyloid in the pathogenesis of AD has been extensively debated over the past several decades [4].

Only recently have clinical, imaging, and biomarker studies in AD patients firmly established the central role of amyloid in disease initiation and progression, and in triggering the spread of tau pathology and neurodegeneration. The development of highly specific PET imaging tools that enable detection and quantitation of the insoluble forms of deposited amyloid and tau have led to ground-breaking longitudinal clinical studies that defined the temporal relationship between the appearance of brain amyloid deposits and the later stages of disease, which are characterized by extensive tau pathology and progressive neurodegeneration [3]. These studies demonstrated that accumulation of amyloid plaque increases during the course of the disease but does not correlate with clinical progression.

While the use of PET imaging tools helped elucidate the temporal relationship among pathological markers of disease, identification of the early trigger of disease pathology and, therefore, a potential AD drug target, remained elusive. The answer emerged from multiple recent studies that identified highly neurotoxic soluble aggregates of amyloid, called Aβ oligomers (oligomers), as upstream drivers of AD pathogenesis that lead to tau hyperphosphorylation and aggregation in NFTs, synaptic injury, and neuronal loss [5,6,7]. Soluble Aβ oligomers further aggregate into insoluble beta sheets, which form amyloid fibrils and plaques that can be detected in patients using amyloid PET imaging tracers [8,9]. Soluble oligomers range from small oligomers, dimers consisting of two monomers, up to dodecamers consisting of twelve monomers, as well as larger Aβ protofibrils, and each oligomer subtype shows a distinct mechanism and pattern of toxicity [5,6].

PET imaging using either amyloid or tau tracers [8,9,10] has further elucidated the evolution of core AD pathologies: amyloid plaques accumulate slowly in the brain and may remain asymptomatic for 20 years or more. NFT pathology first appears in the medial temporal lobe, and only in the presence of a high amyloid burden extends to neocortical regions, propagating along neuronal networks [11,12]. The topography of tau pathology, in particular its progression to the neocortex, correlates closely with cognitive decline and clinical symptoms in AD patients [11]. Studies of biomarkers in CSF of AD patients show a continuous decline in levels of soluble Aβ monomers that parallels cortical amyloid plaque deposition, followed by a gradual increase in phosphorylated tau (p-tau) levels [13,14,15]. These CSF changes precede the appearance of aggregated tau on PET imaging [3,11]. Longitudinal clinical studies confirm the primary and etiological role of oligomer toxicity as the initiator of the pathogenic process in AD and a key driver of disease progression [11].

For decades, the key question in AD drug development has been: “Which species of amyloid is the most toxic and the most relevant as a therapeutic target to slow or stop disease progression? Monomers, soluble oligomers, or insoluble fibrils and plaques?” This question has galvanized the quest for novel therapeutics that target Aβ production, clearance of insoluble plaques and fibrils, or, most recently and successfully, agents that preferentially inhibit the formation of neurotoxic soluble Aβ oligomers or remove them from the brain [16,17].

Clinical data with agents that target Aβ oligomers to varying degrees show that the efficacy of these agents closely correlates with their: (1) selectivity for Aβ oligomers; and (2) degree of oligomer engagement based on their blood–brain barrier penetration and resultant brain drug concentrations. Anti-amyloid agents that have shown clinical or biomarker efficacy in late-stage clinical trials include the anti-amyloid antibodies aducanumab [18], lecanemab (previously called BAN2401) [19], donanemab [20], and gantenerumab [21], and the small molecule oral agent ALZ-801, a prodrug of the active agent tramiprosate [22,23,24]. The four antibodies partially engage and inhibit the toxicity of Aβ oligomers to varying degrees [25,26,27], while the oral highly brain-penetrant ALZ-801 inhibits amyloid monomer misfolding and aggregation, and at the clinical dose fully blocks the formation of all soluble oligomers in the brain [28].

## 2. Soluble Beta Amyloid (Aβ) Oligomers Play an Early Role in Alzheimer ’s Disease (AD) Pathogenesis and Drive Disease Progression

**Figure 1:**
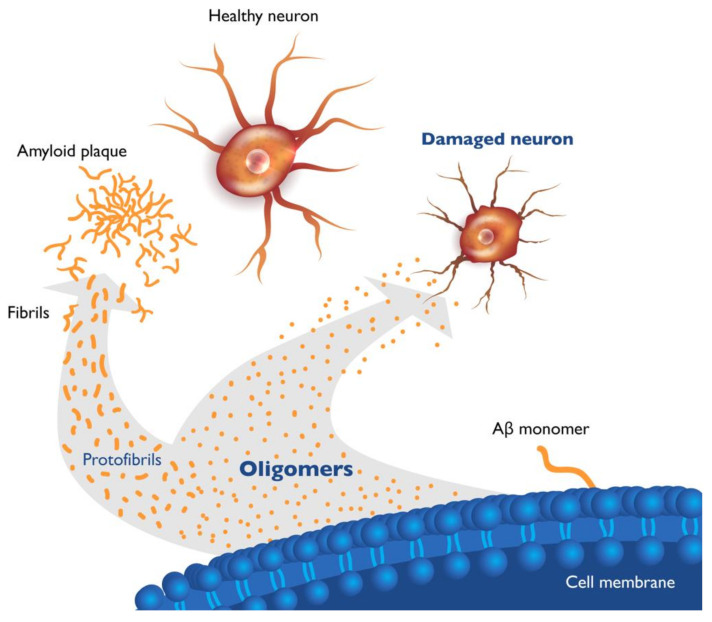
Beta amyloid species in brains of Alzheimer’s patients. Cleavage of amyloid precursor protein in neuronal membranes produces amyloid monomers. Misfolded beta amyloid (Aβ) monomers aggregate into soluble oligomers of various lengths (dimers to dodecamers) and soluble protofibrils (large oligomers). Oligomers further aggregate into insoluble fibrils and plaque. Soluble Aβ oligomers, which are highly toxic to neurons and synapses, are considered upstream triggers of Alzheimer’s disease (AD) pathology.

The Aβ monomer peptide is a proteolytic derivative of the large transmembrane amyloid precursor protein (APP) and is formed by the sequential enzymatic cleavage of APP [29]. Aβ monomers exist as peptides of several lengths, cleave from the neuronal cell membrane, and, in the setting of impaired clearance from the brain caused by aging or genetics, aggregate to form soluble Aβ oligomers of increasing molecular weight [30,31,32], ultimately forming insoluble amyloid fibrils and plaques (Figure 1). The most abundant Aβ monomer in the brain is Aβ40, while the Aβ42 monomer is the most prone to misfolding and aggregation, and is the major form involved in seeding amyloid aggregates and oligomer formation.

Small soluble Aβ oligomers consist of aggregates of two (dimers) to twelve (dodecamers) copies of Aβ monomers. These small oligomers are highly neurotoxic and can further aggregate to form soluble protofibrils (large oligomers) and then insoluble β-pleated sheets forming amyloid fibrils and plaques. As soluble Aβ42 is the predominant form sequestered into plaques, the ratio of soluble Aβ42 to Aβ40 in CSF decreases early in AD, providing a useful diagnostic biomarker of the disease [13,14]. Evaluation of the ratio of soluble Aβ42 to Aβ40 can improve the accuracy of diagnosis of AD and was found to correlate with positivity on amyloid PET scans and changes in hippocampal volume [33]. The formation of insoluble fibrils and plaques appears to be a protective mechanism that mitigates and reduces oligomer toxicity [32,34,35]. Microglia play an important role in the uptake of soluble amyloid, including oligomers, and the formation of compact β-sheets that become the cored dense plaques. This process has been shown to protect adjacent neurons and, by sequestering toxic oligomers into plaques, microglia may play a protective role in AD brains [34,35].

**Figure 2:**
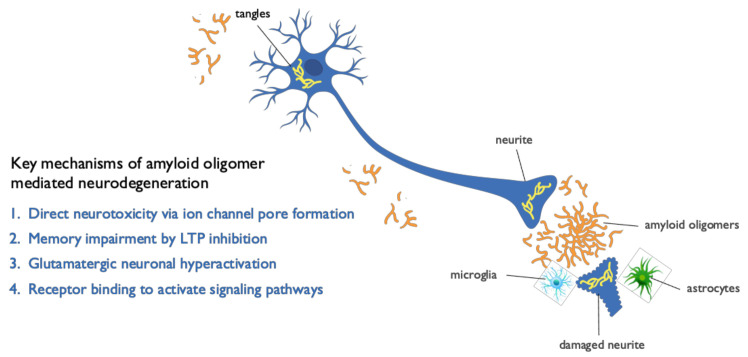
Mechanisms mediating neurotoxicity of amyloid oligomers. Beta amyloid (Aβ) oligomers mediate multiple pathogenic mechanisms in Alzheimer’s disease (AD) that lead to neuronal cell dysfunction and death, including direct synaptic and cell toxicity, neuronal hyperactivation, inhibition of long-term potentiation (LTP), tau hyperphosphorylation, microglial activation, and neuroinflammation.

Aβ oligomers are highly heterogeneous and exhibit a range of sizes, conformations, modes of aggregation, and stages of emergence in AD brain. Correspondingly, different types of oligomers have been shown to elicit distinct mechanisms of neuronal injury [36]. Several pathogenic mechanisms have been identified as responsible for neurodegeneration caused by Aβ oligomers or contributing to their toxicity (Figure 2). Aβ oligomers directly induce mitochondrial dysfunction and oxidative stress, leading to massive calcium influx into brain cells and resultant toxicity [37]. Aβ oligomers have been shown to exert direct neurotoxicity by forming ion channel pores in neuronal membranes that disrupt intracellular calcium homeostasis [37], leading to mitochondrial dysfunction and oxidative stress [38]. In hippocampal tissue slice studies, oligomers were found to affect memory and learning by directly blocking hippocampal long-term potentiation (LTP), the first step of memory formation [39]. In addition, Aβ oligomers trigger a redistribution of critical synaptic proteins and induce hyperactivity in metabotropic and ionotropic glutamate receptors, which result in calcium overload, tau hyperphosphorylation, insulin resistance, oxidative stress, and synaptic loss [40]. Notably, this Aβ oligomer-dependent hyperactivity precedes amyloid plaque formation and is present at early stages of the disease, long before the appearance of clinical symptoms, further supporting the causative role of oligomers in AD pathogenesis [41]. Aβ oligomers also bind to cellular prion protein (PrPC) receptors with high affinity and specificity. The oligomer–PrPC complex with co-activation of the mGluR5 receptor activates intracellular Fyn kinase, leading to calcium disturbances, tau hyperphosphorylation, and disruption of synapses [41,42].

To further understand the contribution of each oligomer species to mechanisms of injury or neurodegeneration, it is important to develop analytical technologies that separate and characterize oligomers of different sizes and conformations in brain and CSF. The development of such oligomer assays in CSF would be a major advance towards the pressing goals in AD therapeutics: (1) an early and more accurate AD diagnosis to enable intervention before development of clinical symptoms; (2) a precision medicine approach to treating patients based on their individual burden of disease; and (3) development of highly targeted and effective anti-oligomer drugs [16,17].

**Figure 3:**
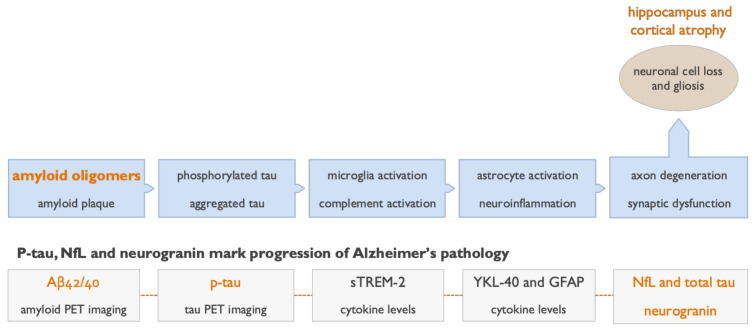
Fluid and imaging biomarkers detect and track progression of pathological processes in Alzheimer’s disease (AD). The core pathologies of AD can now be detected in patients using validated cerebrospinal fluid (CSF) assays for Aβ42, Aβ40, and several isoforms of p-tau. These CSF biomarkers of soluble amyloid and p-tau species indicate a pathological state, while the positron emission tomography (PET) scans show the actual accumulation of insoluble amyloid and tau over time. The toxic downstream effects of amyloid oligomers can be assessed using CSF and plasma biomarkers of neurodegeneration (neurofilament light chain protein (NfL) and total tau) and a biomarker of synaptic injury (neurogranin). Neuroinflammation can be assessed in CSF using the microglial marker (sTREM-2) and astrocytic marker (YKL-40), and in plasma using the astrocytic marker glial fibrillary acidic protein (GFAP) [43]. Insoluble fibrillar amyloid and aggregated tau in NFTs can be visualized and evaluated quantitatively by amyloid and tau PET imaging [8,9,10]. The use of volumetric magnetic resonance imaging (MRI) is well established in AD, accurately detects hippocampal and cortical atrophy reflecting neuronal cell loss and gliosis, and correlates with progression of disease.

Data from recent late-stage AD trials with anti-amyloid antibodies suggest that Aβ neurotoxicity is mediated by soluble amyloid oligomers rather than insoluble aggregates [17]. As soluble Aβ oligomers result in neuronal toxicity via multiple pathways, several potential biomarkers, such as amyloid, tau, neurofilament light chain protein (NfL), neurogranin, neuroglial markers, and inflammatory cytokines [13,14,15], can be used to interrogate the contribution of these pathways to AD progression (Figure 3).

## 3. Genetic Evidence Points to a Central Role of Aβ Oligomers in AD Pathogenesis

Genetic studies in AD patients have identified more than 200 mutations that cause early onset familial AD, and most exhibit autosomal dominant inheritance [44]. Most of these mutations occur in the APP gene, and result in a single biological phenotype characterized by an increased production of Aβ monomers, in particular Aβ42. This amyloid overproduction leads to increased levels of misfolded Aβ42 and its aggregation into toxic amyloid oligomers and plaque, and is associated with AD onset usually before the age of 50.

Some of the rarer mutations lead to elevated levels of soluble amyloid species and early onset of AD symptoms without plaque deposition, supporting the central pathogenic role of Aβ oligomers in AD. The genetic mutations of APP that lead to altered levels of soluble Aβ oligomers include the Osaka, Arctic, and Icelandic mutations [45,46,47,48]. Patients with the autosomal recessive Osaka mutation carry the E693Delta APP variant, which results in enhanced Aβ oligomer production and development of early dementia with minimal plaque pathology on amyloid PET imaging [45]. The other well-known mutation, which has been utilized in the development of the anti-amyloid antibody lecanemab, is the Arctic mutation, caused by the APParc/E693G variant of APP. The Arctic mutation results in increased formation of large soluble oligomers called Aβ protofibrils, low amyloid plaque levels, and early onset AD [46,47]. The low plaque burden associated with this mutation has been confirmed by PET imaging using (11)C-labeled Pittsburgh compound B [47]. In contrast, a protective Icelandic A673T mutation affecting APP, which reduces Aβ monomer production by approximately 30%, results in a four-fold lower risk of AD [48]. The A673T Icelandic mutation reduces both Aβ oligomer burden and fibril levels, resulting in a biological phenotype that protects against development of AD [48].

## 4. The ε4 Allele of Apolipoprotein E (APOE4) Genotype Increases Brain Oligomer Concentration and Accelerates Course of AD

After aging, the APOE4 genotype is the strongest risk factor for late-onset sporadic AD [49]. Patients carrying the ε4 allele of apolipoprotein E (APOE4) show a substantially higher risk for AD with a lower age of clinical onset. Individuals with two APOE4 alleles (APOE4/4 homozygotes) have a 10–30× greater risk for developing AD compared with the APOE3/3 genotype and develop symptoms of AD on average more than a decade earlier [50,51]. The APOE4 genotype is found in approximately 65% of all AD patients, with 10–15% being APOE4/4 homozygotes. The APOE4 genotype is associated with impaired clearance of amyloid from the brain and increased aggregation of Aβ monomers into oligomers [52,53]. AD patients who are APOE4 carriers show several-fold increased concentrations of Aβ oligomers in their brains compared with non-carriers, likely driving the earlier onset and faster progression of AD [54,55,56]. A study of 14 AD patients and 12 age-matched controls that measured Aβ oligomers in the CSF using a surface fluorescence intensity distribution analysis showed that brain levels of Aβ oligomers in AD patients correlated with worse cognitive scores [54]. Several studies have shown that APOE4 carriers, particularly APOE4/4 homozygotes, have a substantially increased burden of Aβ oligomers [55,56]. Levels of Aβ oligomers in brains of AD patients with the APOE4/4 genotype were approximately three-fold higher than in APOE4 non-carriers [55,56].

**Figure 4:**
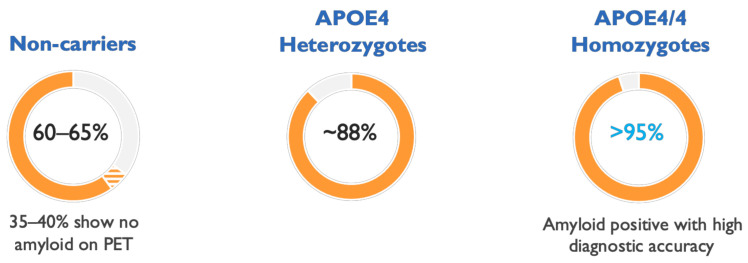
Clinical diagnosis of Alzheimer’s disease (AD) in patients with the APOE4/4 genotype shows high accuracy. The ε4 allele of apolipoprotein E (APOE4) genotype can aid in diagnosis of AD and APOE4/4 subjects show particularly high accuracy of clinical diagnosis. The figure shows rates of positive amyloid positron emission tomography (PET) scans in AD patients by APOE4 genotype. Amyloid PET imaging in APOE4 non-carriers and APOE4 heterozygotes was positive in 65% and 88% of subjects, respectively, increasing to >95% positivity in APOE4/4 homozygotes, based on data from a solanezumab clinical trial in Mild to Moderate AD [57].

In longitudinal AD studies evaluating the natural history of the disease, and in clinical trials, APOE4 carriers show very high rates of amyloid positivity, dramatically improving the diagnostic accuracy of AD in therapeutic trials involving these patients. Amyloid PET data from 390 patients with Mild to Moderate AD showed a mean prevalence of amyloid positivity of 95% in APOE4/4 homozygotes and 88% in APOE4 heterozygotes, compared to ~60% in non-carriers [57], indicating a problematic 40% rate of clinical misdiagnosis in APOE4 non-carriers (Figure 4). Therefore, APOE4 genotyping can be used to substantially improve diagnostic accuracy in AD patients for trials and in clinical practice.

## 5. Clinical and Biomarker Data Show Aβ Oligomers to Be Effective Therapeutic Targets in AD

**Figure 5:**
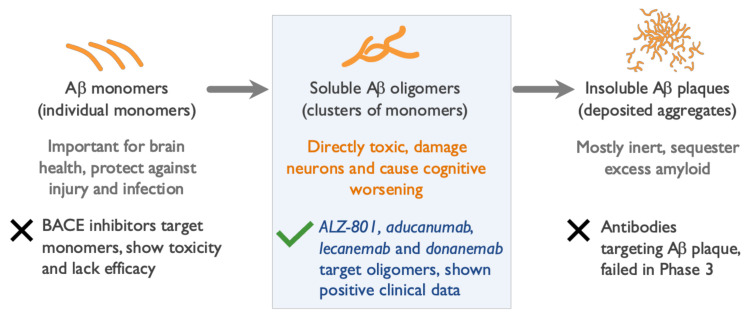
Only agents that target amyloid oligomers show efficacy in Alzheimer’s disease (AD) trials. Among dozens of failures, only four anti-amyloid agents showed positive clinical results in late-stage clinical trials. All four of these agents either selectively inhibit formation of amyloid oligomers or remove oligomers from the brain: the oral tablet ALZ-801 and the intravenously (IV) infused anti-amyloid antibodies aducanumab, donanemab, and lecanemab. Agents that remove or inhibit formation of beta amyloid (Aβ) monomers or clear amyloid plaques failed to show efficacy in AD trials [16,17].

In recent publications, we analyzed the mechanism of action, selectivity, and pharmacokinetics of anti-amyloid agents that were evaluated in large Phase 2 and Phase 3 trials, and summarized their clinical and biomarker effects [16,17]. Anti-amyloid antibodies that predominantly bind Aβ monomers (solanezumab, crenezumab) [58,59] or a mix of monomers and plaques (bapineuzumab) failed to show clinical efficacy in clinical studies. Several types of inhibitors of Aβ monomer production (namely beta-secretase and gamma-secretase inhibitors) failed in Phase 3 trials and were associated with cognitive worsening in AD patients. In contrast, anti-amyloid agents that target Aβ oligomers with various degrees of selectivity have reported statistically significant clinical efficacy in late-stage trials. To date, these include the anti-amyloid antibodies aducanumab, donanemab, and lecanemab, and a small molecule agent, ALZ-801, that fully blocks the formation of Aβ oligomers at the target clinical dose (Figure 5).

The efficacy results of effective anti-amyloid agents parallel their selectivity for Aβ oligomers and preference for oligomers over other amyloid species [25,26,27,28]. Aducanumab and donanemab show similar affinity profiles for Aβ oligomers [27], resulting in similar clinical efficacy, tau PET effects, and rates of vasogenic edema and microhemorrhages [18,20]. Lecanemab, which is more selective for oligomers than aducanumab or donanemab, showed more robust efficacy in the overall study population as well as in APOE4 carriers in a large Phase 2 trial [19,25]. Consistent with its preference for soluble oligomers, and limited clearance of vascular amyloid, lecanemab studies also reported the lowest rates of vasogenic edema among anti-amyloid antibodies (Table 1). Gantenerumab, which binds Aβ monomers as well as oligomers, has shown positive biomarker effects and vasogenic edema, but no clinical efficacy to date [21,60].

The level of p-tau in CSF is the fluid biomarker that shows the best correlation with clinical onset of symptoms and disease progression in AD [61]. In addition, p-tau has also shown significant reductions in response to treatment with anti-amyloid antibodies in recent trials (Table 1). Aducanumab treatment resulted in a significant and dose-dependent reduction in CSF p-tau_181_ in both Phase 3 trials, albeit in a small subset of study subjects [18]. Further support for CSF p-tau_181_ as a biomarker of drug efficacy in AD comes from the lecanemab Phase 2 study, which showed a significant reduction in CSF p-tau_181_ at 78 weeks [19]. Plasma assays for p-tau_181_, and more recently p-tau_217_, are being developed and may provide a convenient blood-based biomarker to evaluate longitudinal changes and drug efficacy in future AD trials [62,63].

In addition to anti-amyloid antibodies, a new class of small molecule agents with improved brain penetration and pharmaceutical properties has advanced to late-stage development in AD. These agents are designed for specific interactions in the Aβ aggregation pathway that lead to inhibition of formation of neurotoxic Aβ oligomers. The most advanced is the orally bioavailable small molecule agent ALZ-801, a conformational modifier of Aβ monomers that fully blocks the formation of Aβ oligomers at the target clinical dose [28,66]. ALZ-801 is currently being evaluated in a Phase 2 AD biomarker trial, and the APOLLOE4 Phase 3 trial in Early AD patients with the APOE4/4 genotype. Another small molecule in clinical development is PQ912, a selective inhibitor of glutaminyl cyclase (GC) that catalyzes formation of a subset of Aβ oligomers. PQ912 inhibits formation of pyroglutamate (pGlu) Aβ oligomers that have shown synaptotoxic, neurotoxic, and proinflammatory activity. In a Phase 2a study, PQ912 showed promising biomarker effects [67] and has advanced to a Phase 2b study.

## 6. Amyloid Plaque Clearance Does Not Correlate with Clinical Efficacy of Anti-Amyloid Agents

The aducanumab data reviewed at the FDA Advisory Committee meeting showed the lack of correlation between plaque reduction on amyloid PET imaging and clinical efficacy [68]. The donanemab Phase 2 study also reported early and significant plaque reduction that did not correlate with clinical efficacy on the primary outcome [20]. Similar findings were observed in the Phase 2 trial of lecanemab, where significant and comparable plaque reduction was observed at the two high doses of the drug, but only the highest dose showed clinical benefit [19], suggesting that the highest dose is necessary to engage Aβ oligomers and drive clinical benefit. Furthermore, the drug withdrawal trial in AD subjects, who completed 78 weeks of lecanemab treatment and were subsequently evaluated off-drug, reported progressive cognitive decline in patients despite a persistently low plaque burden [69]. This indicates that when active treatment is not maintained, the lower exposure levels in brain no longer support engagement of the oligomers, and the toxicity of Aβ oligomers remains unabated and leads to disease progression even in the absence of amyloid plaques.

In summary, there is abundant evidence that clearance of amyloid plaques does not correlate with, or predicts, clinical efficacy. However, plaque removal shows strong and dose-dependent correlation with the side effects of brain edema and microhemorrhage in clinical trials with anti-amyloid antibodies [17]. These data argue against the pathogenic activity of amyloid plaque and further support the role of neurotoxic soluble oligomers as appropriate therapeutic targets [16,17].

## 7. APOE4 Carriers Show Stronger and Less Variable Clinical Efficacy than Non-Carriers

**Figure 6:**
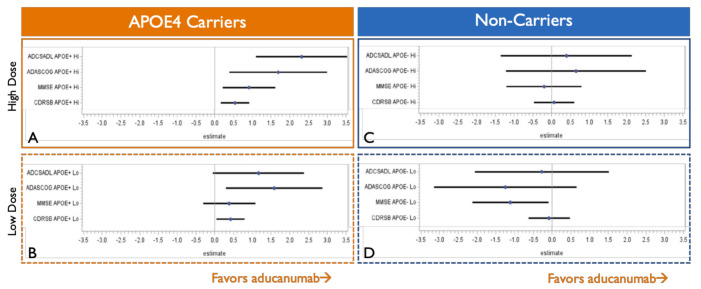
Carriers of ε4 allele of apolipoprotein E (APOE4) drive the overall efficacy of aducanumab in the EMERGE Phase 3 study. Only a single EMERGE Phase 3 trial showed clinical efficacy of aducanumab. Diagrams adapted from the FDA Advisory Committee Briefing Book illustrate point estimate effects of aducanumab in the EMERGE trial on a functional (Alzheimer’s Disease Cooperative Study—instrumental Activities of Daily Living Inventory (ADCSADL)), cognitive (Alzheimer’s Disease Assessment Scale—Cognitive subscale (ADAS-Cog) and Mini-Mental State Examination (MMSE)), and a composite clinical outcome (Clinical Dementia Rating—Sum of Boxes (CDRSB)). Panels (**A**) and (**B**) show effects of high and low doses in APOE4 carriers, and panels (**C**) and (**D**) show effects in APOE4 non-carriers, suggesting that the overall efficacy of aducanumab is derived from the APOE4 carrier population with minimal clinical benefits observed in APOE4 non-carriers [68].

Consistent with the higher burden of amyloid oligomers in the brains of APOE4 carriers, several trials have shown stronger efficacy of anti-amyloid treatments in this group of patients. The positive EMERGE aducanumab Phase 3 trial shows greater clinical benefits in APOE4 carriers [68], which also appear to drive the overall treatment response (Figure 6). In contrast, only minimal and variable clinical efficacy is seen in APOE4 non-carriers. The detailed analysis from the briefing book for the aducanumab FDA Advisory Committee meeting illustrates that in the EMERGE study, APOE4 carriers show greater efficacy on all four clinical outcomes compared with APOE4 non-carriers, with the most pronounced benefit observed in APOE4 patients treated with the highest 10 mg/kg monthly dose.

A similar pattern of efficacy was observed in trials with the oral anti-oligomer agents ALZ-801/tramiprosate and the antibody lecanemab, where APOE4 carriers also showed a substantially larger magnitude of clinical benefit [19,22]. Furthermore, an APOE4 gene dose effect was observed in the tramiprosate Phase 3 trials, where APOE4/4 homozygotes showed the largest efficacy signal, followed by APOE4 heterozygotes and non-carriers [22]. These findings can be explained by the observation that APOE4/4 homozygotes have a three-fold higher oligomer burden in the brain than non-carriers and, therefore, are more responsive to anti-oligomer agents [55,56].

## 8. Oral ALZ-801 Selectively and Fully Inhibits Formation of Aβ Oligomers in Human Brain

The oral agent ALZ-801 specifically and completely blocks aggregation of Aβ monomers into oligomers at the clinical dose without interacting with amyloid plaque [28,70] and, therefore, provides the ideal tool to assess the role of Aβ oligomers in AD pathogenesis and as a drug target for treatment of AD patients. The first task will be accomplished in the ongoing ALZ-801 Phase 2 AD biomarker trial, which is evaluating the full spectrum of plasma and CSF biomarkers, including amyloid, tau, synaptic, neuronal injury, and inflammatory markers, as well as volumetric MRI measures [65]. These biomarkers and clinical endpoints will be evaluated in both APOE4/4 homozygous and APOE3/4 heterozygous subjects with Early AD. The APOLLOE4 ALZ-801 Phase 3 trial in APOE4/4 homozygotes will address the second goal by evaluating cognitive and functional measures, as well as fluid and imaging biomarkers of AD. Since ALZ-801 specifically prevents formation of amyloid oligomers without any interaction with plaques, positive effects of ALZ-801 treatment on clinical measures and biomarkers would provide the crucial evidence for the central role of oligomers in the pathogenesis of AD and open the path for effective disease modification.

ALZ-801 is an optimized prodrug of tramiprosate, which consists of tramiprosate conjugated to the essential amino acid valine, and has been formulated for oral administration with improved absorption and pharmacokinetic properties [24]. The major metabolite of tramiprosate is 3-sulfopropanoic acid, an endogenous molecule in human brain that also inhibits the aggregation of Aβ42 into oligomers by a mechanism of action similar to tramiprosate [66]. In the tramiprosate Phase 3 studies, nausea and vomiting were the main side effects, and plasma levels showed substantial inter-subject variability. For this reason, ALZ-801 was developed to improve gastrointestinal tolerability and to provide consistent plasma levels that resulted in a robust brain penetration of ~40%, and brain levels that fully inhibit formation of amyloid oligomers [24,28]. In a Phase 3 study of patients with Mild to Moderate AD that included all APOE genotypes, tramiprosate showed clinical efficacy in high-risk APOE4 carriers [22], with the largest benefits seen in APOE4/4 homozygotes with Mild AD on the cognitive endpoint as well as two functional outcomes [23].

Phase 1 trials confirmed that an oral dose of 265 mg ALZ-801, administered twice daily, provides plasma exposure equivalent to oral tramiprosate 150 mg twice daily from the tramiprosate Phase 3 trials [24]. Additionally, ALZ-801 oral tablets were well tolerated and showed improved pharmacokinetic properties in healthy volunteers and AD patients. Importantly, treatment with ALZ-801/tramiprosate was not associated with any events of vasogenic brain edema on MRI imaging, consistent with the lack of interaction with insoluble fibrillar and plaque amyloid and clearance of amyloid plaques [70]. In a volumetric MRI study, tramiprosate showed dose-dependent preservation of hippocampal volume compared with a placebo, which supports the disease-modifying effects of ALZ-801/tramiprosate treatment [64]. The utility of hippocampal and cortical volumetric measures as markers of neurodegeneration is especially relevant in APOE4/4 homozygotes, who show significantly accelerated atrophy [65]. These measures will be applied in the ongoing Phase 3 trial of ALZ-801 in homozygous APOE4/4 subjects with Early AD.

## 9. Conclusions

The convergence of data from genetic studies and preclinical mechanistic studies, and the promising results of recent clinical trials, provides ample evidence for the central role of amyloid oligomers in AD pathogenesis and their importance as a therapeutic target for disease modification. There is a renewed sense of optimism and urgency for the late-stage candidates in the AD pipeline, as neurotoxic amyloid oligomers became the first therapeutic target for disease modification with positive clinical and biomarker data. For the first time in history, an opportunity to slow or even stop and prevent this devastating disease is on the horizon.

The anti-amyloid antibodies as a group show similar degrees of modest efficacy, with the additional burden of monthly or more frequent intravenous administrations, the occurrence of vasogenic edema and microhemorrhage due to interaction with insoluble fibrillar and plaque amyloid, and the resultant removal of vascular amyloid plaque. Lecanemab, which is more selective for amyloid oligomers, also shows more substantial cognitive benefits with a lower incidence of vasogenic edema; however, it requires twice monthly administration. Therefore, there is an urgent need for highly selective inhibitors of Aβ oligomer formation or toxicity that can efficiently cross the blood–brain barrier and achieve sustained brain levels to block or prevent the persistent insult of oligomers. Due to the progressive and chronic nature of AD, patients also need therapeutic options compatible with long-term treatment, in particular favorable safety and convenient dosing. Oral ALZ-801 satisfies these criteria, since it has shown robust brain penetration, promising clinical and brain imaging effects at the target clinical dose, and favorable long-term safety and tolerability.

Results from Phase 2 and 3 trials of ALZ-801 in Early AD are highly anticipated to provide evidence of efficacy and to adjudicate the role of Aβ oligomers in AD pathogenesis. An ongoing Phase 2 AD biomarker study is evaluating effects of ALZ-801 on CSF and plasma biomarkers and brain MRI volumetrics in APOE4 carriers (NCT04693520). The confirmatory APOLLOE4 Phase 3 trial of ALZ-801 in APOE4/4 homozygotes with Early AD (NCT04770220) was recently initiated, and will include assessments of clinical outcomes, brain volumes by MRI, and a rich battery of plasma and CSF biomarkers.

A large body of evidence now shows the unique and unparalleled toxicity of soluble Aβ oligomers and establishes their central role in AD pathogenesis. In addition, recent trial results that utilize several distinct therapeutic approaches confirmed Aβ oligomers as effective therapeutic targets for disease modification in AD. These developments bring new hope to Alzheimer’s patients and their families, with the potential for approval of treatments that can slow or arrest clinical decline and even prevent the disease in the near future.

## Figures and Tables

**Table 1:** Engagement of oligomers drives clinical and biomarker effects of anti-amyloid agents. To date, four anti-amyloid agents have shown both positive clinical effects, as well as correlation with fluid or imaging biomarkers: the intravenous antibodies aducanumab, lecanemab, donanemab, and the oral tablet ALZ-801. Aducanumab and lecanemab significantly reduced cerebrospinal fluid (CSF) levels of p-tau_181_, and aducanumab and donanemab showed significant effects on tau positron emission tomography (PET) imaging. ALZ-801/tramiprosate significantly decreased hippocampal atrophy assessed by volumetric magnetic resonance imaging (MRI) measurements [16,64,65].

Clinical & Biomarker Profile	*Biogen* Aducanumab IV Infusion 10 mg/kg Monthly	*Eli Lilly* Donanemab IV Infusion 1400 mg Monthly	*Eisai* Lecanemab IV Infusion 10 mg/kg Twice per Month		*Alzheon* ALZ-801/Tramiprosate Oral Tablet 265 mg Twice Daily
Selectivity for Oligomers *	+	+	++		+++ Blocks oligomer formation
**Study Population**	Early AD All genotypes	Early AD All genotypes	Early AD All genotypes	Early AD APOE4 carriers	Mild AD APOE4/4 homozygotes
**Cognition** ADAS-cog (% benefit vs. placebo)	27% *p* = 0.0097	32% *p* = 0.04	47% *p* = 0.017	84% Not reported	125% *p* = 0.0001
**Function** CDR-SB (% benefit vs. placebo)	22% *p* = 0.012	23% *p* = NS	26% *p* = NS	60% Not reported	81% *p* = 0.0197
**CSF p-tau_181_** (% benefit vs. placebo)	15%	Not reported	13%		Ongoing biomarker study
**Imaging Biomarkers**	Significant decrease in tau PET signal	Significant decrease in tau PET signal	Increase in hippocampal atrophy (7.6%, not significant)		Significant decrease of hippocampal atrophy
**Brain Edema** (% vs. placebo)	35% (42% in APOE4)	27%	10%	15%	0%

* Based on published relative selectivity data [25,26,27,28].
